# Controlling Sodium Titanate Crystal Size to Improve Wettability and Early Osseointegration of Titanium Implants: Insights from an Animal Model

**DOI:** 10.3390/jfb16080283

**Published:** 2025-08-01

**Authors:** Saray Fernández-Hernández, Javier Gil, Marta Sanjuán-Álvarez, Ignacio Sanz, Mariano Herrero-Climent, Aritza Brizuela-Velasco

**Affiliations:** 1 DENS-ia Research Group, Faculty of Health Sciences, Miguel de Cervantes European University, 47012 Valladolid, Spain; abrizuela@uemc.es; 2 Bioengineering Institute of Technology, Facultad de Medicina y Ciencias de la Salud, Universidad Internacional de Catalunya, C/Josep Trueta s/n, 08195 Sant Cugat del Vallès, Spain; 3 Biomimetics Oral Biomaterials and Interfaces (BOBI), Department Ciencia e Ingeniería de Matariales, Escola d’Enginyeria Barcelona Est, Universitat Politècnica de Catalunya, c/Eduard Maristany 16, 08029 Barcelona, Spain; javier.gil.mur@upc.edu; 4 Faculty of Health Sciences, Miguel de Cervantes European University, 47012 Valladolid, Spain; msanjuan@uemc.es; 5 Section of Graduate Periodontology, Faculty of Dentistry, Complutense University, 28040 Madrid, Spain; ignaciosanz@ucm.es; 6 Porto Dental Institute, Av. de Montevideo 810, 4150-518 Porto, Portugal; dr.herrero@herrerocliment.com

**Keywords:** dental implants, sodium titanate crystal, bone-to-implant contact

## Abstract

The thermo-chemical treatment of dental implants leads to the formation of sodium titanate crystals on their surface. When in contact with blood, these crystals dissolve and trigger an ionic exchange cascade, resulting in the formation of a calcium apatite layer. This study, carried out both in vitro and in an animal model, aimed to determine whether the cooling rate of the treatment affects the size of the deposited crystals, and whether this in turn influences wettability and early bone-to-implant contact (BIC). A total of 50 dental implants and 50 titanium discs were treated using four different cooling rates, along with a control group. Crystal size was analyzed on implant surfaces using scanning electron microscopy, and wettability was assessed on titanium discs using a goniometer. Finally, the implants were placed in the tibiae of 13 rabbits, and histological analysis was performed after three weeks to compare BIC among groups. Results suggest that a cooling rate of 75 °C/h produces smaller sodium titanate crystals, which are associated with significantly improved surface wettability and a higher percentage of bone-to-implant contact after 3 weeks of healing (*p* < 0.05).

## 1. Introduction

The process of osseointegration of a dental implant has similarities with the primary healing of a bone fracture, the fundamental difference being the presence of an alloplastic material at the edges of the newly formed bone tissue [1].

Due to their excellent biocompatibility and corrosion resistance, dental implants are usually made of commercially pure titanium (cpTi, grade 1–4) or the titanium alloy Ti-6Al-4V (grade 5). While cpTi has a higher corrosion resistance and a more favorable biological behavior, Ti-6Al-4V offers a higher mechanical strength and is therefore better suited for narrow diameter or load-bearing implants. These material differences also affect the surface properties; cpTi generally has a higher surface energy and wettability, while alloyed surfaces generally require further modification to achieve comparable hydrophilicity [2].

The most widely used technique to prepare the implant site—conventional rotary drilling—causes damage to the extracellular bone matrix. Consequently, during implant placement, the surface is exposed to extracellular fluids rich in proteins, enzymes, cytokines, and bone growth factors [3], such as albumin, fibrinogen, immunoglobulins G and M, fibronectin, and osteopontin.

At the same time, platelet aggregation begins almost immediately after surgical drilling. Upon platelet degradation, various growth factors are released into the surrounding environment, including PDGF (Platelet-Derived Growth Factor), TGF-β (Transforming Growth Factor Beta), VEGF (Vascular Endothelial Growth Factor), IGF-1 (Insulin-like Growth Factor 1), as well as BMP-2 and BMP-7 (Bone Morphogenetic Proteins). Some of these factors stimulate osteoblast proliferation and differentiation, while others promote angiogenesis and regulate neovascularization, a key process during the regeneration phase [4].

These proteins bind to the implant surface molecules through weak interactions, such as Van der Waals forces. It has been shown that protein adsorption is strongly influenced by the chemical and morphological properties of the implant surface [5]. The surface wettability plays a key role here, as more hydrophilic surfaces have a higher surface energy and enable more effective contact with biological fluids. This increased interaction area not only increases the amount of adsorbed proteins, but also improves their orientation and conformation, thereby maintaining their biological functionality. Consequently, highly wettable surfaces exhibit greater molecular and cellular affinity, improve biocompatibility, and facilitate the initiation of the healing process [6].

It is important to note that this initial protein adsorption on the implant surface triggers osteoblast migration and subsequent adhesion through the recognition of a specific amino acid sequence: arginine–glycine-aspartic acid (RGD). This is followed by a phase of focal adhesion in which proteins are recruited to stabilize the osteoblasts and promote cytoskeletal reorganization and morphological changes, which allow cell survival and full functionality [7]. The result is the production of a new osteoid matrix by the osteoblasts, which is initially only weakly mineralized, and later calcified by the incorporation of hydroxyapatite crystals anchored in a three-dimensional network of type I collagen. Finally, the tissue is remodeled under functional load by a mechanotransduction-mediated process controlled by osteocytes [8,9,10,11,12].

One of the most commonly used histologic parameters to evaluate osseointegration in preclinical studies is the bone-to-implant contact (BIC), defined as the percentage of the implant surface in direct contact with bone without the interposition of soft tissue, as seen in histologic sections. The BIC provides an objective quantification of the quality and quantity of bone integration and has become an important outcome measure for the validation of new implant surfaces. Several studies have shown that a rough implant surface roughness achieved by specific additive or primarily subtractive modification techniques can improve BIC and ultimately osseointegration compared to machined surfaces [13,14,15]. Although this variable is of great interest, it inevitably requires the explantation of the implants once they are osseointegrated in order to perform a histological analysis. As a result, studies to evaluate this variable are performed in animal models, with the rabbit tibia model being one of the most commonly used [13].

In recent years, however, surface modification strategies have increasingly focused on the nanoscale. These nanostructured modifications aim to create bioactive surfaces that promote accelerated bone formation on the implant. In general, such treatments are effective in improving early BIC, although not necessarily in achieving higher levels of BIC values at conventional integration times (e.g., 8 weeks). Nevertheless, accelerating osseointegration is of particular clinical interest in certain surgical procedures such as immediate loading, post-extraction implants, or medically compromised patients. Different bioactive treatments have different biological effects [3,16]. These include hydroxylation of the surface to reduce the contact angle and improve protein adsorption, anodic oxidation to increase surface porosity and roughness, and thus promote cell adhesion, and hydrofluoric acid etching, whereby fluoride increases osteogenic and osteoinductive activity.

Another strategy involves incorporating calcium phosphate particles into the surface to stimulate platelet activation and osteoblast migration [17,18,19,20,21,22]. In this context, the thermochemical treatment of Kokubo consists of applying an alkaline solution (NaOH) to the titanium surface, followed by heat treatment. This process leads to the formation of sodium titanate crystals on the surface, which dissolve on contact with blood or saliva. When sodium ions are released, the surface becomes negatively charged and attracts calcium ions, followed by phosphate ions. The result is the formation of a crystalline calcium phosphate layer (hydroxyapatite) that mimics the mineral phase produced by osteoblasts during the initial phase of bone formation. In implant systems with calcium phosphate coatings already applied, part of the coating may be lost during insertion under high insertion torques. In contrast, with thermochemical treatment (e.g., VEGA and VEGA+; Klockner, Escaldes-Engordany, Andorra), the hydroxyapatite layer forms in situ after bone insertion of the implant due to the ion exchange mechanism described.

Recently, our research group published an in vitro study in which the influence of the crystal size of sodium titanate on osteoblastic behavior was investigated. The results supported that smaller crystals are associated with improved cell adhesion and higher alkaline phosphatase activity. However, due to the inherent limitations of the in vitro design, it was not possible to determine whether these improvements also translate into favorable histological outcomes for osseointegration [23].

The present experimental study, combining in vitro and animal model trials, aims to determine the influence of variations in cooling rate during thermochemical treatment of titanium surfaces on the size of sodium titanate crystals formed. The subsequent objective is to assess the relationship between the crystal size and the surface wettability. The primary objective of the animal model study is to evaluate whether smaller crystal sizes are associated with improvements in early osseointegration, using BIC as the main outcome variable.

In summary, the null hypothesis of the present study is that changes in cooling rate during thermochemical treatment of titanium implants do not significantly affect sodium titanate crystal size, surface wettability, or early bone-to-implant contact (BIC) in vivo.

## 2. Materials and Methods

In order to achieve the study objectives, two complementary experimental designs were carried out: an in vitro experiment and an animal model study. The in vitro experiment aimed to determine the influence of the cooling rate during a thermochemical surface treatment on a sample set of titanium implants, particularly with regard to the size of the sodium titanate crystals formed. The differences in wettability between the different crystallographic configurations were then analyzed. In the animal model study, implants with different crystal sizes were used to investigate their influence on bone-to-implant contact (BIC) during an early osseointegration period (3 weeks).

### 2.1. In Vitro Experiment Study

**Figure 1:**
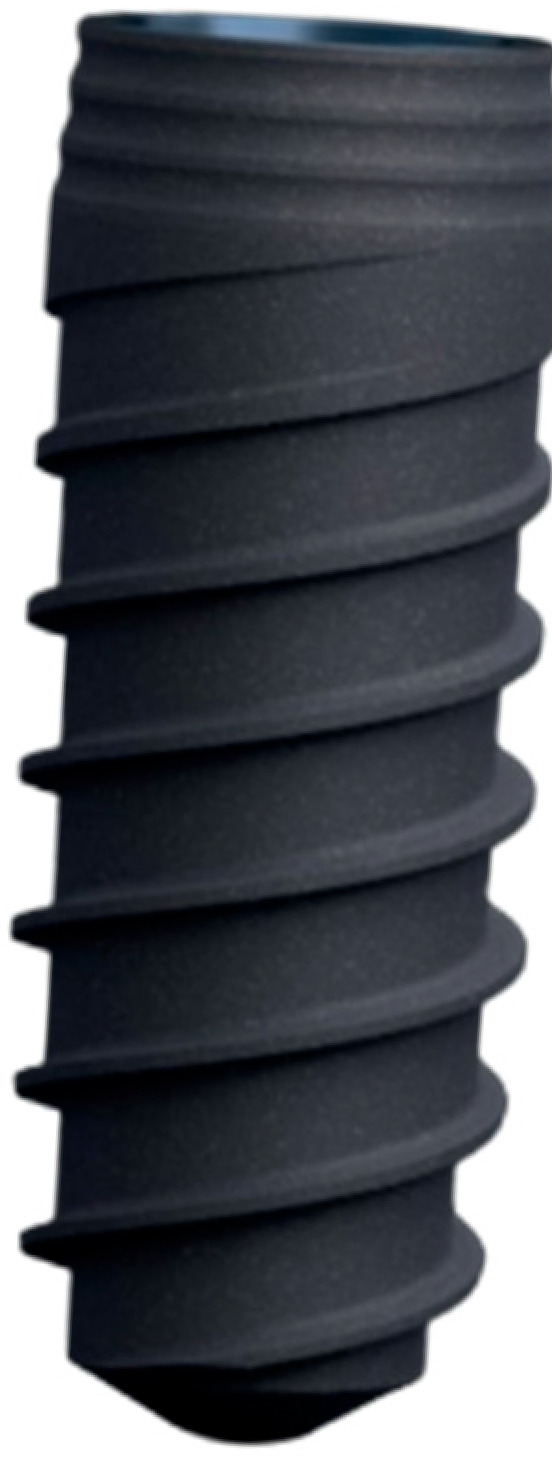
Bone-level, internal connection implant used in this study (VEGA, Klockner, Escaldes-Engordany, Andorra).

Fifty VEGA dental implants made of commercially pure grade 3 titanium, with a diameter of 3.5 mm and a length of 8 mm, were donated by Klockner Dental Implants (Escaldes-Engordany, Andorra) for this study (Figure 1). These implants are identical in all structural, compositional, and design aspects to the conventional implants used in clinical practice. Ten implants served as controls, while the remaining forty underwent the same thermochemical treatment, differing only in the cooling rate (*n* = 10 implants per group).

The thermochemical modification process followed the methodology described by Kokubo et al. [11], with slight adjustments. Each sample was immersed in 10 mL of a 5 M sodium hydroxide solution (NaOH) and incubated at 60 °C for 24 h. After alkali treatment, the samples were thoroughly rinsed with distilled water and dried at 40 °C for a further 24 h. Thermal treatment was then carried out in a tube furnace, where the samples were heated to 600 °C at a rate of 5 °C/min, where they remained for one hour. A continuous flow of high purity argon gas (99.99%) was used throughout the process to prevent oxidation of the titanium.

To evaluate the influence of the cooling rate, 10 implants per group were cooled in the furnace at different rates: 20 °C/h, 50 °C/h, 75 °C/h, and 115 °C/h. Another group with untreated implants (*n* = 10) served as a control.

The surface morphology was analyzed with a high-resolution scanning electron microscope (TESCAN CLARA UHR SEM, Brno, Czech Republic) at an accelerating voltage of 15 kV and with several working distances. The crystal size of the sodium titanate layer formed under different cooling conditions was quantified with ImageJ software (version 1.54d, National Institutes of Health, Bethesda, MD, USA) using the 2 nm resolution of the microscope.

In addition, grade 3 titanium disks (8.5 mm diameter, 4 mm thickness) were prepared for wettability analysis. These disks were subjected to the same surface and thermochemical treatments as the implants, including the four cooling rates and the control condition (*n* = 7 per group). The contact angle was measured using an OCA 11 goniometer (Dataphysics, Filderstadt, Germany), with one measurement per sample using 2 µL of distilled water.

### 2.2. Animal Model Study

A total of 13 adult male New Zealand white rabbits, each weighing approximately 3.5–4 kg, were used for this study. The experimental protocol followed the “Animals in Research: Reporting In Vivo Experiments” (ARRIVE) guidelines to ensure reproducibility and transparent reporting. Ethical approval was granted by the Animal Research Ethics Committee of the Universitat Autònoma de Barcelona (approval code 2013-05 of 04-26-2013, dated 15 February 2022).

All animal procedures were conducted at the animal facility of the Facultat de Veterinària, Universitat Autònoma de Barcelona (Edifici V, Animalari UAB), in accordance with institutional and European ethical guidelines.

Four implants were placed per rabbit—two per tibia—placed in the proximal region close to the epiphysis on the medial surface with a distance of 6 mm distance between implants. (*n* = 52). In summary, 10 implants were used for each test group (with the 4 different cooling rates), and 12 implants were used in the control group (without thermo-chemical treatment).

Although no formal power analysis was performed using statistical software, the sample size was based on previous studies with similar designs and outcome measures [24,25], which demonstrated statistically significant differences between groups under comparable conditions. This approach was considered sufficient to ensure 80% statistical power at a 5% significance level, while also adhering to the ethical guidelines of the 3Rs principle (Replacement, Reduction, and Refinement).

The allocation of implants to each site was performed using a randomized block design to ensure balanced distribution among the five groups (four test groups and one control). A computer-generated randomization list was used to assign each implant position across the 52 available sites (two per tibia), ensuring that no animal received more than one implant from the same group.

All surgeries were performed by an experienced surgeon who was blinded to the study objectives and unaware of implant group assignment; only the principal investigator (JG) was aware of the experimental group allocation.

All animals were operated on by the same surgical team, on the same day, and housed under identical conditions before surgery and during the osseointegration period.

General anesthesia was achieved through an intramuscular injection combining dexmedetomidine (Dexdomitor; Ecuphar, Barcelona, Spain), ketamine (Ketamidor; Karizoo, Barcelona, Spain), and buprenorphine (Bupaq; Richter Pharma, Wels, Austria). During the surgical procedure, anesthesia was sustained via inhalation of isoflurane (Isoflo; Zoetis, Madrid, Spain) administered at a constant concentration of 1–2%. Additionally, local anesthesia was applied at the surgical site by infiltrating articaine hydrochloride with epinephrine (40/0.01 mg/mL) (Ultracain; Laboratorios Normon, Madrid, Spain).

**Figure 2:**
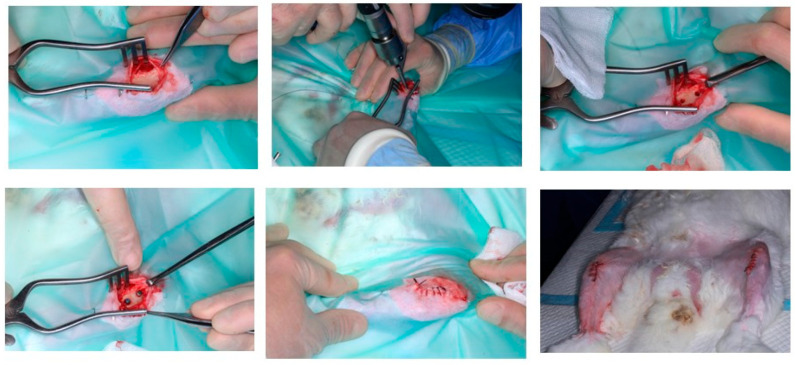
Step-by-step sequence of the surgical procedure performed in this study.

A single surgical incision was performed along the medial aspect of each tibia, followed by elevation of a full-thickness soft tissue flap. Implants were positioned in the predetermined sites using the drilling sequence recommended by the manufacturer and inserted manually, ensuring that the insertion torque did not exceed 35 N·cm (Figure 2). Surgical closure was completed in layers using 4-0 Vicryl sutures (Johnson & Johnson International, New Brunswick, NJ, USA).

No adverse events or complications were observed in any of the animals during the 3-week osseointegration period, and all subjects remained suitable for analysis. Animals were housed under standard laboratory conditions, which included unrestricted access to food and water and a 12 h light/dark cycle.

At the end of the observation period, rabbits were sedated with a combination of dexmedetomidine (Dexdomitor; Ecuphar, Barcelona, Spain) and diazepam (Valium; Roche Farma, Madrid, Spain), followed by euthanasia by intravenous administration of sodium pentobarbital at a dose of 100 mg/kg. After euthanasia, both tibias were extracted and bone segments containing the implants were harvested for histomorphometric evaluation.

The harvested samples were subjected to dehydration with a graded ethanol series (70–100%), followed by infiltration with increasing concentrations of ethanol and glycol methacrylate (Technovit 7200 VLC, Heraeus Kulzer, Wehrheim, Germany). Polymerization was completed by heating the samples at 37 °C for 24 h. Central longitudinal cuts of about 200 µm thickness were made with a precision band saw and reduced to about 40 µm by mechanical micropolishing (Exakt Apparatebau, Norderstedt, Germany) with 1200 and 4000 grit silicon carbide abrasive paper (Struers, Copenhagen, Denmark). The sections were then stained using the Levai-Laczkó technique for histological analysis.

High-resolution images of the implant–bone interface were acquired using a scanning electron microscope (SEM) in backscattered electron mode. The region of interest was the cortical bone in the coronal region of the implant, and therefore, the apical region was excluded from the analysis, as some implants in the sample had apical anchorage due to anatomical location and bone availability, while others did not. Histomorphometric measurements were performed with a high-resolution scanning electron microscope focusing on the cortical bone area in the coronal part of the implant. Image analysis was performed with ImageJ software (version 1.54d, National Institutes of Health, Bethesda, MD, USA) which allowed differentiation in grayscale and quantification of mineralized bone in direct contact with the implant surface. The bone-to-implant contact (BIC) ratio was calculated and expressed as a percentage.

Descriptive statistics were performed for the in vitro and animal model assessments, including measures of central tendency (mean) and dispersion (standard deviation). For the primary dependent variable (BIC), data normality was assessed using the Shapiro–Wilk test prior to performing inferential analysis. Statistical comparisons were then performed using Student’s *t*-tests, one-way ANOVA, and Tukey’s post hoc tests to assess significant differences between groups (implants treated with different cooling rates and different sodium titanate crystal sizes) with a significance level of α = 0.05.

## 3. Results

**Figure 3:**
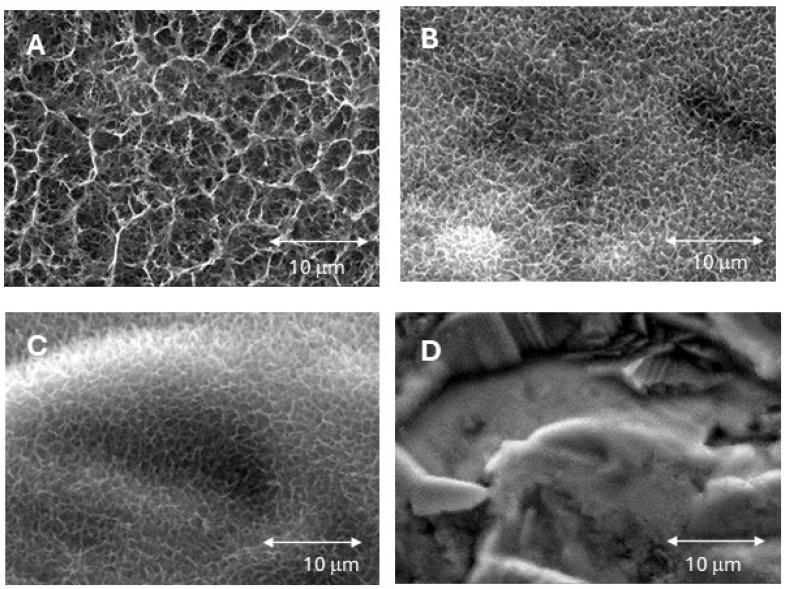
(**A**) Surface of biomimetic dental implant with cooling rate at 20 °C/h. (**B**) Surface of biomimetic dental implant cooling rate at 50 °C/h. (**C**) Surface of biomimetic dental implant cooling rate at 75 °C/h. (**D**) Surface of biomimetic dental implant cooling rate at 115 °C/h.

Figure 3 shows the surfaces of the biomimetic dental implants that were exposed to different cooling rates. As the cooling rate increases, a reduction in crystal size can be observed. At the highest cooling rate, the surface appears to lack a clearly defined crystallization and instead exhibits an amorphous phase, although some very fine crystals can still be observed in certain areas.

The crystal sizes (in micrometers) measured with the image analysis software integrated in the scanning electron microscope are listed in Table 1.

At a cooling rate of 20 °C/h, the resulting crystal size was almost twice as large as that obtained at a cooling rate of 75 °C/h. However, as mentioned above, a cooling rate of 115 °C/h resulted in a surface that was almost free of crystalline structures and exhibited an amorphous state with crystal sizes of approximately 0.12 μm.

**Figure 4:**
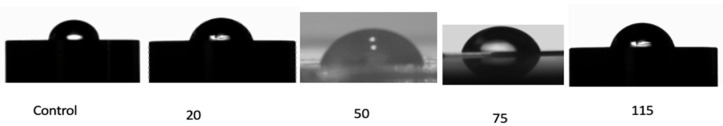
Representative images of water droplets on samples exposed to different cooling rates (20, 50, 75, and 115 °C/h) during thermochemical treatment, together with an untreated control. Differences in droplet shape illustrate variations in contact angle, reflecting changes in surface wettability associated with the size of sodium titanate crystals formed.

Table 2 contains the results of the wettability analysis showing the contact angle measured on each treated surface. As the crystal size decreased, the contact angle also decreased, except at the highest cooling rate (115 °C/h), which resulted in an increased angle of up to 80° (Figure 4).

A contact angle below 90° indicates strong adhesion forces between the liquid and the surface, i.e., the liquid is “attracted” to the surface and spreads on it. Therefore, all treated surfaces can be considered hydrophilic, although the surface treated at a cooling rate of 75 °C/h exhibited the highest hydrophilicity with a contact angle of 69° ± 6.

Table 3 shows the results of the bone-to-implant contact (BIC). Statistically significant differences were observed for all cooling rates compared to the control group (*p* < 0.005), except for the highest cooling rate.

In particular, the cooling rate of 75 °C/h resulted in a bone-to-implant contact area that was almost twice as large as that of the control group.

## 4. Discussion

The aim of the present experimental in vitro and animal model study was to determine the influence of the size of sodium titanate crystals deposited on the surface of dental implants for human use on the rate of early osseointegration, assessed by histologic analysis (BIC).

Changing the cooling rates during thermochemical surface treatment of the experimental implant groups resulted in the deposition of sodium titanate crystals of different sizes. The results of the implant samples in this study, which showed that higher cooling rates tended to result in smaller crystal sizes, are consistent with those reported in a previous in vitro study on titanium discs [23]. In fact, both studies agree that a cooling rate of 75 °C/h is optimal to obtain smaller crystals, while rates higher than 115 °C/h led to the formation of amorphous structures that lack biomimetic activity.

This phenomenon is explained by the principles of solid-state diffusion processes: faster cooling leads to undercooling, which promotes the formation of numerous crystal nuclei, as the critical radius for their stability is smaller than under less undercooled (i.e., more slowly cooled) conditions [26]. These numerous nuclei grow into smaller crystals, resulting in a surface consisting of many small sodium titanate crystals instead of fewer, larger crystals, as can be clearly seen in Figure 3.

Furthermore, when the implants are cooled at 115 °C/h, there is not enough time for crystal formation and an amorphous layer is formed. This amorphous layer is less stable, degrades faster under physiological conditions, and does not have the osteoinductive effect characteristic of crystalline sodium titanate [27].

The smaller crystal size achieved at a cooling rate of 75 °C/h results in a larger specific surface area in contact with the physiological environment and causes a higher negative surface charge due to the dissolution of sodium cations. This, in turn, improves the adsorption of osteoprogenitor proteins such as fibronectin, and promotes faster overall osseointegration [28,29,30].

In addition, this fine crystalline morphology improves wettability, as shown by the reduced contact angles observed with titanium discs in the present study. Increased wettability improves biological performance by promoting greater blood contact, protein adsorption, cell migration, and ultimately faster tissue formation.

It is important to note that the wettability tests could not be performed directly on the implant samples, which is a limitation of the present study. However, the crystal sizes obtained at each cooling rate showed no statistically significant differences (*p* < 0.05) between the implants and the corresponding discs, and were consistent with the results of the previous in vitro experiment [23].

In this previous study, improved osteoblast adhesion and increased alkaline phosphatase activity were observed in the samples with smaller sodium titanate crystals (cooling rate 75 °C/h), suggesting accelerated osseointegration. This hypothesis is confirmed in the present animal model study (New Zealand rabbits), in which the BIC values measured at an early osseointegration stage (3 weeks) were also higher in implants treated at this optimal cooling rate.

It is important to emphasize that the osseointegration period evaluated can be considered early, especially considering that standard osseointegration times are around 8 weeks in humans and around 6 weeks in animal models such as rabbits [25,31].

In this context, it should be noted that osseointegration is a time-dependent process. In conventional microrough surfaces (e.g., those treated by sandblasting), a decrease in implant stability is usually observed between the second and fourth week after placement, which is due to resorption of the initial bone in contact with the surface. Subsequent bone formation restores secondary stability, with acceptable BIC values usually reached around the eighth week [14,32].

In contrast, surfaces modified with alkaline and thermal treatments show higher BIC values at earlier time points [21,33,34,35]. This effect is attributed to the formation of a calcium phosphate apatite layer, which results from the dissolution of sodium titanate crystals in contact with blood and plasma in the implant bed and has a similar chemical composition to the mineral phase of the bone. As a result, it does not trigger inflammatory responses and instead promotes favorable osteoblastic and protein responses [36,37].

**Figure 5:**
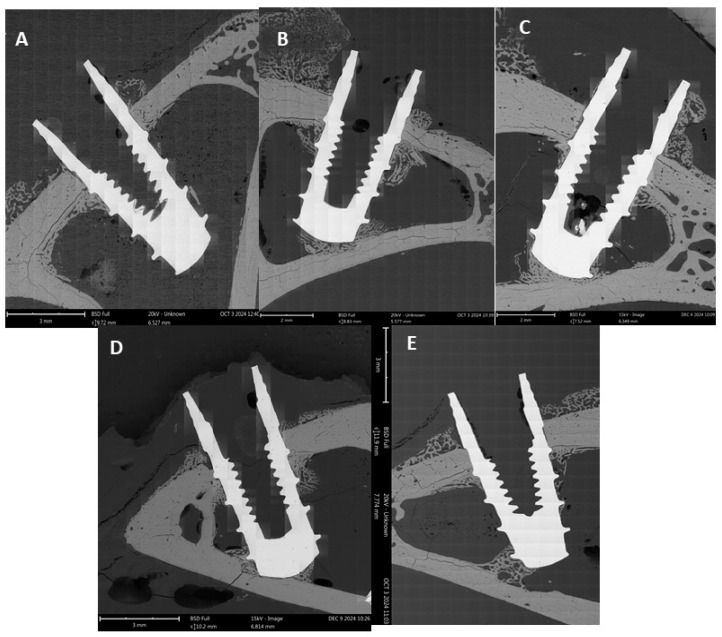
Backscattered electron images of resin-embedded, undecalcified sections showing the bone–implant interface in the cortical region. (**A**) Control implant without thermochemical treatment. (**B**) Implant treated with cooling rate of 20 °C/h. (**C**) Cooling rate of 50 °C/h. (**D**) Cooling rate of 75 °C/h. (**E**) Cooling rate of 115 °C/h.

The effect observed in our study (Figure 5)—an increase in early indicators of successful and accelerated osseointegration on bioactive surfaces within the first four weeks—has been confirmed in numerous animal studies [33,34,38,39], although not all used BIC as the primary outcome variable. Some studies examined bone area density (BAT) or bone density in a defined region of interest (ROI), typically from the first implant thread to a depth of 1 mm.

Furthermore, our results are consistent with clinical studies evaluating this type of surface, where secondary implant stability is often used as the main outcome variable [32,40,41,42,43].

However, it is important to recognize that several studies have shown that between the eighth and twelfth week, BIC values become comparable between bioactive and standard surfaces (microrough sandblasted and acid-etched surfaces) [21,32,33,44,45,46,47,48,49,50].

Essentially, as observed in our study, bioactive surfaces are expected to achieve higher BIC values at earlier osseointegration time points, but not necessarily higher BIC values than non-bioactive surfaces at conventional healing times—an aspect that could not be controlled in our experimental design.

However, due to their ability to promote earlier secondary stability by accelerating osseointegration, the clinical indications for such surfaces are obvious: poor bone quality, immediate or early loading, post-extraction implants, and patients with limited healing capacity (e.g., diabetes, immunosuppression, advanced age, and osteoporosis) [51,52].

It is true that the bone availability in the rabbit tibia is limited, which may lead to differences in apical cortical anchorage between implants. This variability could influence implant stability. However, the randomized allocation of implant positions in this study should mitigate such effects by compensating for anatomical differences between groups. Despite its limitations, the rabbit tibia remains a widely used and accepted model in preclinical implant research due to its practicality and biological relevance [13].

The use of backscattered electron images on resin-embedded sections is suitable for the quantitative analysis of bone-to-implant contact (BIC), but does not provide information on the presence of inflammatory cells, the quality of newly formed bone, or general biological activity. This is a limitation of the present study and should be investigated in future studies using complementary histological or immunohistochemical techniques.

Based on the results obtained, the null hypothesis of the present study must be rejected, as the cooling rate during thermochemical treatment significantly affects both surface wettability and early bone-to-implant contact (BIC) by modulating the sodium titanate crystal size.

## 5. Conclusions

Within the limitations imposed by the nature of the measurements and the experimental design of this study, it can be concluded that a cooling rate of 75 °C/h during the thermochemical treatment of dental implants is associated with the deposition of smaller sodium titanate crystals. This, in turn, appears to influence both the increase in surface wettability and the improvement in the percentage of bone-to-implant contact (BIC) observed in an animal model during the early stages of osseointegration.

## Figures and Tables

**Table 1:** Descriptive statistics (mean and standard deviation) of the sodium titanate crystal sizes (µm) formed after applying the different tested cooling rates.

Cooling Rate (°C/h)	Crystal Size (μm) Mean ± SD
20	0.77 ± 0.34
50	0.48 ± 0.25
75	0.39 ± 0.20
115	0.12 ± 0.09

**Table 2:** Descriptive statistics (mean ± standard deviation) of the contact angle values (in degrees) obtained in the wettability test for each surface condition, corresponding to the different sodium titanate crystal sizes produced by the cooling rates tested.

Cooling Rate (°C/h)	Contact Angle (°) Mean ± SD
Control (without treatment)	91 ± 3
20	87 ± 7
50	72 ± 8
75	69 ± 6
115	80 ± 9

**Table 3:** Mean and standard deviation of the BIC values for the different experimental groups. Asterisks indicate statistically significant differences (*p* < 0.005) compared to the control group.

Cooling Rate (°C/h)	BIC (%) Mean ± SD
Control (without treatment)	37.5 ± 5.6
20	59.8 ± 7.0 *
50	68.2 ± 5.2 *
75	73.5 ± 9.0 *
115	41.3 ± 9.2

## Data Availability

The original contributions presented in the study are included in the article; further inquiries can be directed to the corresponding author.
